# KGCAK: a K-mer based database for genome-wide phylogeny and complexity evaluation

**DOI:** 10.1186/s13062-015-0083-4

**Published:** 2015-09-16

**Authors:** Dapeng Wang, Jiayue Xu, Jun Yu

**Affiliations:** CAS Key Laboratory of Genome Sciences and Information, Beijing Institute of Genomics, Chinese Academy of Sciences, Beijing, 100101 PR China; Stem Cell Laboratory, UCL Cancer Institute, University College London, London, WC1E 6BT UK; University of Chinese Academy of Sciences, Beijing, 100049 China

**Keywords:** Database, K-mer, Genome phylogeny, Genome complexity

## Abstract

**Background:**

The K-mer approach, treating genomic sequences as simple characters and counting the relative abundance of each string upon a fixed K, has been extensively applied to phylogeny inference for genome assembly, annotation, and comparison.

**Results:**

To meet increasing demands for comparing large genome sequences and to promote the use of the K-mer approach, we develop a versatile database, KGCAK (http://kgcak.big.ac.cn/KGCAK/), containing ~8,000 genomes that include genome sequences of diverse life forms (viruses, prokaryotes, protists, animals, and plants) and cellular organelles of eukaryotic lineages. It builds phylogeny based on genomic elements in an alignment-free fashion and provides in-depth data processing enabling users to compare the complexity of genome sequences based on K-mer distribution.

**Conclusion:**

We hope that KGCAK becomes a powerful tool for exploring relationship within and among groups of species in a tree of life based on genomic data.

**Reviewers:**

This article was reviewed by Prof Mark Ragan and Dr Yuri Wolf.

## Implementation

### Introduction

Over the past few decades, phylogenies have often been built from defined evolutionarily-conserved gene families and occasionally from sequences of whole genomes. Two categories of methodology are used for such phylogeny building, distinguished by whether sequence-alignment is applied or not, and the alignment-free methodology has become more popular [1]. K-mer technique has been shown to be exceedingly effective in a variety of genomic applications, including genome assembly, motif discovery, repetitive sequence identification, and genome complexity assessment [2–6]. One of the other notable applications is to construct sequence trees from K-mer arrays, especially for coding regions and protein sequences [7, 8]. With the rapid accumulation of large genomic datasets in diverse species, the need for an easy-to-use database that stores and visualizes processed K-mer based data is obvious, and it is especially useful for biologists to explore phylogenetic relationship among a large collection of species that are publicly available and adequate for further mining. Toward this end, we establish KGCAK database (K-mer-based Genome Composition Analysis Knowledgebase) that exploits within-species K-mer arrays and allows cross-species comparisons, for phylogeny building. The original sequences include a variety of sequences, including those from whole genomes, cDNAs, CDS, proteins, and non-coding RNAs, where K varies from 2 to 10 for nucleotide and from 1 to 5 for protein sequences. In addition, we categorize various genomes based on the context and feature of K-mer arrays in a user-friendly manner, including statistics and frequency distribution of K-mer array with a fixed K and unique ratio of K-mer array with variable K. We provide several in-depth functions, such as 2D or 3D plotting for the investigation of linear correlations between multiple pre-defined measures stemming from the above-mentioned calculations. Our objective is to build a platform that captures features of genome sequences and turns digital K-mer arrays from genomes into easy-to-understand and visualized data from a comparative genomics perspective.

### Methods and Materials

Genomes and gene annotations were acquired from Ensembl (ftp://ftp.ensembl.org/ and ftp://ftp.ensemblgenomes.org/), Phytozome (http://www.phytozome.net/) and NCBI (ftp://ftp.ncbi.nlm.nih.gov/genomes) genome databases. For the shared genomes between Phytozome and Ensembl, only one representative was used. K-mer array is defined as an array that comprises frequencies or occurrence probabilities based on K-length strings from all possible combinations of characters (nucleotides or amino acids) and arranged in a fixed order. Two state-of-the-art tools, *Tallymer* and *Jellyfish* are used to estimate the uniqueness of K-mer and to create K-mer arrays for each condition, respectively [6, 9]. For three indexes of *Tallymer*, "unique ratio" is defined as the fraction of number of K-mers that occur only once, "nonunique ratio" is defined as the fraction of the number of non-unique K-mers for each K merely accounting for the events, and "nonuniquemulti ratio" is defined as the fraction of number of non-unique K-mers for each K with the consideration of occurrence number of the same K-mer. We construct phylogenetic trees using popular *CVtree* methods [8, 10]. In brief, we begin with K-mer arrays from nucleotide (4^K^) or amino acid sequences (20^K^) to be compared and compute correlations and distances between pairwise arrays (vectors) to build up distance matrices. In this process, we use background random corrections to reduce noise only for K > =4. We offer three methods from *PHYLIP*, including *Fitch*, *Kitsch* and *Neighbor* [11], for tree topology prediction based on distance matrix and *the Newick Utilities* for viewing the trees [12]. The final data for "Genomic parameter" and "K-mer statistics" are imported into separate MYSQL tables, and the intermediate data for "Tree construction", "Frequency distribution" and "Uniqueness ratio" were stored in the hierarchical and structural folders.

#### Functionality and Performance

**Fig. 1:**
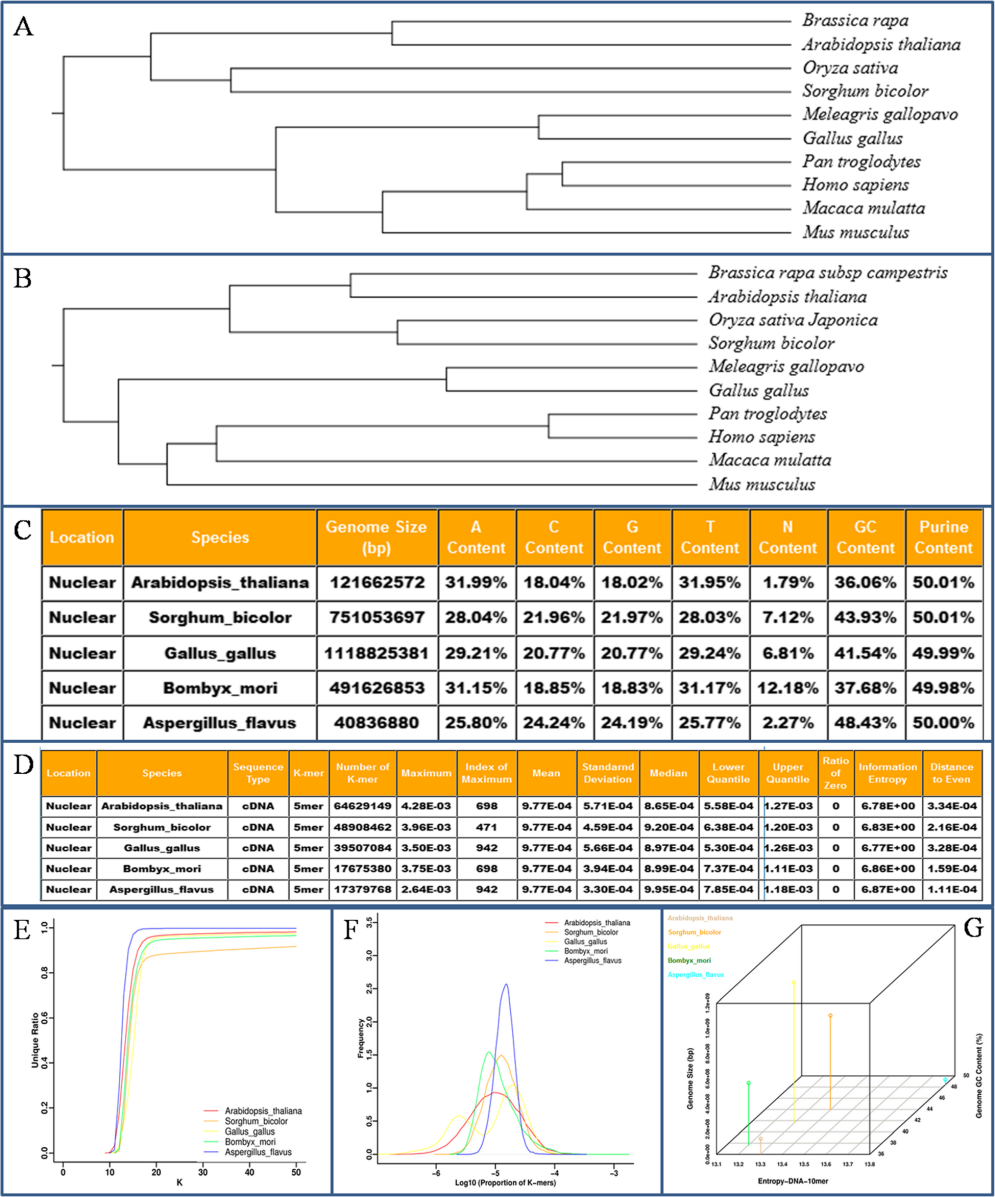
An brief overview for KGCAK functionality. **a** A tree built from 5-mer protein sequences of nuclear genomes based on kitsch method. **b** A tree built from 5-mer protein sequences of mitochondrial genomes based on kitsch method. **c** An example for genomic parameter result from DNA sequences in five nuclear genomes. "A Content", "C Content", "G Content", "T Content", "GC Content", and "Purine Content" represent the percentages of nucleotide A, C, G, T, G + C and A + G in T + C + A + G of genomic sequences; N Content means percentage of nucleotide N in T + C + A + G + N of genomic sequences. **d** An example for K-mer statistics from cDNA sequences in five nuclear genomes in terms of 5-mer. In particular, "Information Entropy" is defined as the Shannon information entropy calculated from a K-mer array and the formula is H = −∑P_i_log(P_i_), where P_i_ is frequency of each K-mer. "Distance to Even" indicates the summary of square of difference between individual element and global average value in the K-mer array. **e** An example for uniqueness ratio from DNA sequences in five nuclear genomes. **f** An example for frequency distribution from DNA sequences in five nuclear genomes in terms of 8-mer. **g** An example for genome-complexity-3D compared between entropy-DNA-10mer, genome GC content, and genome size from five nuclear genomes

To access data stored in this database, we provide three basic modes: "comparison mode", to compare multiple species at the same time, "search mode" to view the data, and "browse mode" to explore data in a single genome fashion. For the use of "comparison mode", users are allowed to choose the number of species to be compared from 5, 10, and 15 on the first page. Subsequently, the users are able to enter the full Latin names of the species of interest in the textbox. In addition, there are seven functions, three genome locations ("Nuclear", "Mitochondrial" and "Plastid"), five genomic elements ("DNA", "cDNA", "CDS", "ncRNA" and "Protein") and five (1–5 for proteins) or nine (2–10 for nucleotides) lengths of K-mers. We describe these functions in details as follows. "Tree construction" gives the illustrations of tree topologies as well as the trees in *Newick* format with reference to the three popular tree reconstruction approaches from K-mer arrays from different genomes for a comparative purpose (Fig. 1a). The users can build trees using not only data of nuclear genomes but also those of organellar genomes, and furthermore, users are able to compare similarity and difference between trees generated from the different genomic elements of the same or close-related species (Fig. 1b). "Genomic parameter" compares some fundamental genome parameters, such as gene structure, number and length as well as genome composition; for example, GC content and purine content can be compared at three levels: whole genome, mRNA and ncRNA (Fig. 1c). "K-mer statistics" shows arithmetic statistics of K-mer array among genomes, such as "Number of K-mer", "Maximum", "Index of Maximum", "Mean", "Standard Deviation", "Median", "Lower Quantile", "Upper Quantile", "Ratio of Zero", as well as "Shannon information entropy" [5] (Fig. 1d). "Uniqueness ratio" draws three images exhibiting features of unique ratio, nonunique ratio and nonunique multi-ratio on K, revealing compound effects of genome size, repetitive sequences and genome complexity, especially at the macro-evolutionary scale, and such features are helpful in determining optimal K parameters for species-specific genome assembly. Figure 1e exemplifies that the curve of unique ratio approaches the maximum in *Sorghum bicolor* at a lower rate than that does in *Aspergillus flavus*, meaning that *Aspergillus flavus* has a relatively simpler genome composition or architecture, lacking repetitive regions [6]. "Frequency distribution" plots K-mer frequency distribution that concerns fraction and absolute number, where peak number and shape reflect presence or absence (or abundant or rare) appearance of particular K-mer sequences as species-specific signatures, especially when a set of genomic elements or species are compared. Figure 1f shows that *Gallus gallus* exhibits bimodal distribution, which behaves in a quite different manner from other four species, in particular, as compared with *Aspergillus flavus* that has a single sharp peak [3]. "Genome complexity-2D" and "genome complexity-3D" carry two-way or three-way comparisons for some chosen genomic parameters by drawing scatter plots along with linear regression analysis and point plots for exploring genome structural features that show genome complexity among purpose-defined genomes (Fig. 1g). In the future, we expect to update the data and improve its performance by adopting data compression and multi-threading computation techniques demanded by *big data* and high-speed processing when larger number of K-series of additional genomes are included.

## Availability and requirements

**Project name:** KGCAK

**Project home page:**http://kgcak.big.ac.cn/KGCAK/

**Operating system(s):** Linux

**Programming language:** PHP, Mysql, R and Perl

**Other requirements:** No

**License:** No restrictions for academic users.

**Any restrictions to use by non-academics:** licence needed

## Reviewers comments

### Reviewer 1: Prof Mark Ragan

As the authors indicate, methods based on counts or frequency distributions of k-mers are becoming popular in bioinformatics and computational molecular bioscience. This includes the two specific application areas addressed by this manuscript: phylogenetics, and the evaluation of genome complexity. It is not unreasonable, then, than an online data resource might support research in these areas.

The authors have adequately addressed other concerns that I raised in regard of an earlier draft of this paper.

The range of k values is much too limited for comparison of whole genomes, including the inference of genome trees. The maximum k needs to be much larger. It is unclear why k = 1 is included for protein.

*Authors’ response: It has been reported that the phylogeny tends to be stable when K = 5 for protein sequences in most cases* [10, 13]*. We noticed that some of the tools for similar purpose adopt K = 10 for nucleotide sequences* [7] *but still keep in mind that some improvements may be brought in when a larger K value is used especially for the large genome and repeat-rich sequences. In fact, we have tried to do this for nucleotide sequences with K up to 15, but the data files take up too much space since the K-mer array is rather long, especially under the condition that we included a large number of genomes and genomic elements, which exceed our current computer storage capacity. To tackle this problem, we plan to introduce compression technology for the updated version in the future. For K = 1 in protein, we reckon that it is a good starting point since the K-mer array has 20 elements already.*

### Reviewer 2: Dr Yuri Wolf

Wang, Xu and Yu present a database of k-mers, KGCAK, derived from the completely sequenced genomes of viruses, bacteria, archaea and eukaryotes. Unfortunately, some details of the data processing are vague in the text and the utility of the offered database is dubious.

The authors say that they acquired the genome sequences from Ensembl, Phytozome and NCBI genome databases (p. 4 of the manuscript), but give the links only to the generic websites. The NCBI FTP site (where most of the prokaryotic genomes in KGCAK seem to originate) keeps the genomic data in at least three different locations, offering genomes in different states of completeness and curation, in different formats etc. The authors state that "only one representative was kept for the same genomes that occur multiple times in different data sources" (p. 4), but no details were given regarding how the "sameness" was established ("Methanococcus_jannaschii", "Methanocaldococcus jannaschii str. DSM 2661" and "Methanocaldococcus_jannaschii_DSM_2661_uid57713" are "the same", but it is not easy to find using a naive comparison of text strings). For eukaryotes no information is given regarding the treatment of multiple mRNAs and proteins derived from the same locus (almost 97000 protein sequences, or over 3 times the number of genes, are currently annotated in the genome of *H. sapiens*).

*Authors’ response: We have revised the sentence to include more details in the website. In terms of the representative for the same genomes, we only refer to either Phytozome or Ensembl and the sameness was established based on the identical species name. For other species unique to either database, we did not take this step. We have clarified this point in the relevant sentences. As to the isoform issue, we used all transcripts that are well annotated in the genomes as input to calculate K-mer parameters and perform downstream analysis, and we intend to include relevant information as much as possible from one genome to present its overall property.*

At the user's side, the database website offers limited tools for comparison and analysis. Links to k-mer trees lead to trees, reconstructed using arbitrary sets of species that do not demonstrate anything in particular. Comparisons of distributions are only visual (no quantitative measures of distribution similarities are given) and us smoothed curves to show essentially discrete data. The user interface is not well-designed and sometimes puts an unnecessary burden on the user (e.g. the "Comparison" page prompts the user to enter species names that have to be spelled in a particular manner, with "Escherichia coli" not being a valid choice).

*Authors’ response: To avoid confusion, we have removed the function for reconstructing trees using one user specified genome and another 9 random selected genomes. As to the comparison of distributions, we have added a function that performs Kolmogorov-Smirnov test to compare K-mer frequency distribution. To facilitate proper input for species names, we have placed a link on each relevant page to all genome/species names defined in this database. Consequently, users are able to check the correct species name before further actions. Since we have included many different subspecies or strains for the same species, the full name (sometimes a bit longer) is used.*

Given the above, it is difficult to imagine that the KGCAK database will be actively used by the research community for any meaningful purpose, with a possible exception of as a teaching aid.

The main issue - that the KGCAK database doesn't seem to solve any particular problem or serve any discernible purpose - remains unchanged.

*Authors’ response: Using this database, we expect to address a couple of crucial theoretical and practical questions in two dimensions such as genomic elements and species. For example, to understand the link between genome components and organism complexity, the robustness of the phylogenetic trees built from K-mer arrays, and the similarity and difference between genomic parameters in a wide variety of lineages.*

## Abbreviations

CDS: The coding sequence
ncRNA: The non-coding RNA
